# Supplementary material for: An open-source framework for large-scale, flexible evaluation of biomedical text mining systems
Source: J Biomed Discov Collab. 2008 Jan 29;3:1. doi: 10.1186/1747-5333-3-1 (PMC2276192; doi:10.1186/1747-5333-3-1)
Supplement: Additional file 1 — Evaluation platform source code. The additional file contains the software evaluation framework discussed in this paper. The most current release can be downloaded from SourceForge.net [1]. See the accompanying README file for instructions on installation and use. Also included in the distribution is a collection of UIMA wrappers for some commonly used BioNLP tools and annotated corpora. [file 1747-5333-3-1-S1.zip › bionlp_uima_repository-1.0.1/docs/api/src-html/edu/uchsc/ccp/util/nlp/document/GenericDocument.html]

```
001    package edu.uchsc.ccp.util.nlp.document;
002    
003    import java.io.PrintStream;
004    import java.util.ArrayList;
005    import java.util.HashMap;
006    import java.util.List;
007    import java.util.Map;
008    
009    import edu.uchsc.ccp.util.nlp.annotation.TextAnnotation;
010    
011    public class GenericDocument {
012    
013            private String titleText;
014            private String abstractText;
015            private String documentID;
016        private int documentCollectionID;
017        private List<String> secondaryDocumentIDs;
018        
019        /* This map should eventually take the place of the secondaryDocumentIDs list. This way, you can distinguish between different types of IDs*/
020        private Map<String, String> otherDocumentIDs;
021    
022            private String documentText;
023            private List<DocumentSection> documentSections;
024    
025            private List<TextAnnotation> annotations;
026    
027            public GenericDocument(String documentID) {
028            otherDocumentIDs = new HashMap<String, String>();
029            secondaryDocumentIDs = new ArrayList<String>();
030                    this.documentID = documentID;
031                    annotations = new ArrayList<TextAnnotation>();
032        }
033        
034        public GenericDocument() {
035            otherDocumentIDs = new HashMap<String, String>();
036            annotations = new ArrayList<TextAnnotation>();
037            secondaryDocumentIDs = new ArrayList<String>();
038        }
039    
040        /**
041         * Retrieve any other identifiers associated with this document
042         * @return
043         */
044        public Map<String, String> getOtherDocumentIDs() {
045            return otherDocumentIDs;
046        }
047        
048        /**
049         * Insert a new document ID (other than the primary ID)
050         * @param idType
051         * @param id
052         */
053        public void addOtherDocumentID(String idType, String id) {
054            otherDocumentIDs.put(idType, id);
055        }
056        
057            public List<TextAnnotation> getAnnotations() {
058                    return annotations;
059            }
060    
061            public void setAnnotations(List<TextAnnotation> annotations) {
062                    this.annotations = annotations;
063            }
064    
065            public void addAnnotation(TextAnnotation annotation) {
066                    this.annotations.add(annotation);
067            }
068    
069            @Deprecated
070            public String getAbstractText() {
071                    return abstractText;
072            }
073            @Deprecated
074            public void setAbstractText(String abstractText) {
075                    this.abstractText = abstractText;
076            }
077            @Deprecated
078            public String getTitleText() {
079                    return titleText;
080            }
081            @Deprecated
082            public void setTitleText(String titleText) {
083                    this.titleText = titleText;
084            }
085    
086            public String getDocumentID() {
087                    return documentID;
088            }
089    
090            public void setDocumentID(String documentID) {
091                    this.documentID = documentID;
092            }
093    
094            public List<DocumentSection> getDocumentSections() {
095                    return documentSections;
096            }
097        
098        public void addDocumentSection (DocumentSection ds) {
099            if (documentSections != null) {
100                documentSections.add(ds);
101            } else {
102                documentSections = new ArrayList<DocumentSection>();
103                documentSections.add(ds);
104            }
105        }
106    
107            public void setDocumentSections(List<DocumentSection> documentSections) {
108                    this.documentSections = documentSections;
109            }
110    
111            public String getDocumentText() {
112                    return documentText;
113            }
114    
115            public void setDocumentText(String documentText) {
116                    this.documentText = documentText;
117            }
118    
119        public int getDocumentCollectionID() {
120            return documentCollectionID;
121        }
122    
123        public void setDocumentCollectionID(int documentCollectionID) {
124            this.documentCollectionID = documentCollectionID;
125        }
126    
127        @Deprecated
128        public List<String> getSecondaryDocumentIDs() {
129            return secondaryDocumentIDs;
130        }
131    
132        @Deprecated
133        public void setSecondaryDocumentIDs(List<String> secondaryDocumentIDs) {
134            this.secondaryDocumentIDs = secondaryDocumentIDs;
135        }
136        
137        @Deprecated
138        public void addSecondaryDocumentID(String secondaryDocID) {
139            this.secondaryDocumentIDs.add(secondaryDocID);
140        }
141    
142        public void printDocument(PrintStream ps) {
143            ps.println("==============================================");
144            ps.println("DOCUMENT: " + this.documentID);
145            ps.println("TITLE: " + this.titleText);
146            ps.println("ABSTRACT: " + this.abstractText);
147            ps.println("--------- ANNOTATIONS ---------");
148            for (TextAnnotation ta : this.annotations) {
149                ta.printAnnotation(ps);
150            }
151        }
152        
153    }
```
